# Adipose-derived, autologous mesenchymal stem cell therapy for patients with post-COVID-19 syndrome: an intermediate-size expanded access program

**DOI:** 10.1186/s13287-023-03522-1

**Published:** 2023-10-05

**Authors:** Ridhima Vij, Hosu Kim, Hyeonggeun Park, Thanh Cheng, Djamchid Lotfi, Donna Chang

**Affiliations:** 1 Hope Biosciences Research Foundation, 16700 Creek Bend Dr., Sugar Land, TX 77478 USA; 2 Hope Biosciences, Sugar Land, TX 77478 USA

**Keywords:** Autologous, Adipose-derived mesenchymal stem cells, Intravenous, Post-COVID-19 syndrome, Efficacy

## Abstract

**Background:**

Evolving mutations of the novel coronavirus continue to fuel up the pandemic. The virus affects the human respiratory system along with other body systems, causing several sequelae in the survivors of the disease, presented as post-COVID-19 syndrome or long-COVID-19. This protocol utilized Hope Biosciences’ autologous, adipose-derived mesenchymal stem cells (HB-adMSCs) to evaluate safety and efficacy of HB-adMSC therapy to improve signs and symptoms associated with post-COVID-19 syndrome.

**Methods:**

Ten eligible subjects with post-COVID-19 syndrome were enrolled in the program for a duration of 40 weeks who received 5 intravenous infusions of 2 × 10^8^ autologous HB-adMSCs each at week 0, 2, 6, 10 and 14 with a follow-up at week 18 and end of the study at week 40. Safety assessments included incidence of adverse and serious adverse events along with the laboratory measures of hematologic, hepatic, and renal function. Efficacy was examined by quality-of-life assessments, fatigue assessments, Visual analog scale (VAS) of symptoms and monitoring of respiration and oxygen saturation rates.

**Results:**

VAS scores and Fatigue Assessment scores (FAS) showed significant improvements post-treatment (*P* = 0.0039, ES = 0.91) compared to baseline. Respiration rates and oxygen saturation levels that were within the normal range at the baseline remained unchanged at the end of the study (EOS). Paired comparison between baseline and EOS for short-form-36 health survey questionnaire (SF-36) scores also showed improved quality-of-life with significant improvements in individual SF-36 evaluations. Mostly mild AEs were reported during the study period with no incidence of serious AEs. Also, no detrimental effects in laboratory values were seen.

**Conclusions:**

The results of the expanded access program indicated that treatment with autologous HB-adMSCs resulted in significant improvements in the signs and symptoms associated with post-COVID-19 syndrome as assessed by VAS and FAS scores. Additionally, improvements in the patients’ quality-of-life as demonstrated using SF-36 scores that also showed significant improvements in individual scaled scores. Overall, administration of multiple infusions of autologous HB-adMSCs is safe and efficacious for improvements in the quality-of life of patients with post-COVID-19 syndrome.

*Trial registration*: Clinical trial registration number: NCT04798066. Registered on March 15, 2021. (https://clinicaltrials.gov/ct2/show/NCT04798066?term=hope+biosciences&cond=Post-COVID-19+Syndrome&draw=2&rank=2).

**Supplementary Information:**

The online version contains supplementary material available at 10.1186/s13287-023-03522-1.

## Introduction

Coronavirus Disease 2019 (COVID-19), known to be caused by highly contagious Severe Acute Respiratory Syndrome CoronaVirus-2 (SARS-CoV-2), with the first reported cases in Wuhan, China in December 2019, and was soon recognized as a global pandemic in March 2020 [1]. The most common clinical manifestations of COVID-19 include mild or moderate respiratory symptoms that can quickly lead to more severe complications such as acute respiratory distress syndrome (ARDS), pneumonia, cardiac arrhythmia, multiple organ damage and even death [2]. Pulmonary fibrosis risk is greatly increased in infected patients and may persist even after infection is resolved [3]. In addition to pulmonary symptoms, there is evidence that COVID-19 could also affect the central nervous system, causing post-COVID clinical manifestations like headache, nausea, vomiting, and can potentially lead to neurological diseases [4], resulting in a number of sequelae in patients who survive the disease, severely affecting their quality-of-life. These sequelae have been presented as post-COVID-19 syndrome, and the patients are described as COVID-19 long-haulers [5].

The expression of post-COVID-19 syndrome can be as long as three weeks from the onset of the first symptoms to beyond 12 weeks. Generally, this group of patients can be divided into those who may have serious sequelae (such as thromboembolic complications) and those with non-specific clinical manifestations, often dominated by fatigue and shortness of breath [6]. In either case, due to lack of effective and long-term treatment options, quality-of-life of COVID-19 survivors is negatively affected. Currently, there is no specific therapeutic intervention to successfully improve residual post-infection clinical manifestations in these COVID-19 long-haulers. Available treatment options include anti-viral drugs, anti-inflammatory immune mediated therapies, antibiotics, systemic corticosteroids, and convalescent plasma therapy [7–11]; however, these methods only offer supportive therapy with limited to no effect on transmission or the ongoing long-lasting symptoms. Therefore, there is an urgent need to find effective methods of prevention, treatment, and/or agents to help regulate the damaged immune system, thereby ameliorating residual effects following COVID-19 infection.

Globally, mesenchymal stem cells have become a promising tool for the treatment of autoimmune and infectious diseases, by offering protection against immune attack associated damage along with reparative mechanisms. MSCs are known for their ability to regulate the immune system and inhibit inflammation and cytokine storm [12]. Tissue repair by MSCs has been shown to occur both directly and through the release of paracrine factors [13]. MSCs release anti-inflammatory and antiapoptotic molecules and hence, may protect damaged tissues [14]. MSCs have also been shown effective in acute and chronic inflammatory lung conditions by suppressing the immune response and, possibly, by differentiating into type II alveolar epithelial cells in the repair process [15, 16]. Inhibition of T-cell proliferation is a key immunomodulatory feature of MSCs [17], along with their ability to dampen the immune response and attenuate secondary injury mechanisms [18, 19]. However, safety and clinical efficacy of MSC therapy in treatment of post-COVID-19 sequelae is yet to be determined.

Previously, several studies had demonstrated the clinical benefit of MSC-based therapies in treatment of patients with severe COVID-19 [20–24]. In a recent systematic meta-analysis, Qu et al. demonstrated the potential of MSC therapy associated with improvements in clinical outcomes as well as reduction in mortality in patients hospitalized with COVID-19. They also suggested the potential ability of MSC therapy to reduce the recovery time as well as long-term complications associated with SARS-CoV-2 infection [25]. While other studies implicate the potential benefit of MSC-based therapies for the sequelae followed by COVID-19 infection, the current study is the first one to the best of our knowledge, to illustrate clinical efficacy in reducing signs and symptoms in the patients with post-COVID-19 syndrome. This expanded access protocol utilized Hope Biosciences’ autologous, adipose-derived mesenchymal stem cells (HB-adMSCs) with an aim to evaluate safety and efficacy of HB-adMSCs in the improvement of signs and symptoms associated with post-COVID-19 syndrome. Based on the immunomodulatory properties of MSCs known to inhibit cell-mediated immune inflammatory responses and MSC endogenous regenerative properties, we hypothesize that adMSC therapy would reduce pathological symptoms associated with post-COVID-19 syndrome. Patient-derived autologous stem cells present a safe option because they allow multiple treatments without risk of immune rejection, which could arise from allogeneic cell therapies. Moreover, to measure consistent efficacy results across the study population, fresh, undifferentiated, culture-expanded HB-adMSCs were employed in the current study.

## Methods

### Study design and participants

This is an expanded access study designed to evaluate efficacy and safety of autologous HB-adMSCs in improving the signs and symptoms associated with post-COVID-19 syndrome. A screening period (up to 28-days) was used to collect and analyze all screening data and to decide whether the screened subject is eligible to participate. Ten eligible subjects, who passed screening, received the investigational product: multiple infusions each with 200 million autologous, adipose-derived MSCs. Each subject received a total of 5 infusions for the treatment duration of 14 weeks that included Infusion 1 (Week 0), Infusion 2 (Week 2), Infusion 3 (Week 6), Infusion 4 (Week 10) and Infusion 5 (Week 14) with a follow-up at week 18 and end of the study at Week 40. The study was approved by the Western Institutional Review Board (WIRB) located in Olympia, Washington, and conducted in accordance with Good Clinical Practice guidelines and the Declaration of Helsinki. All participants provided written informed consent. All procedures for this expanded access protocol were performed at Hope Biosciences Research Foundation.

### Patient eligibility

#### Inclusion criteria

(1) aged 18–65 years; (2) a positive SARS-COV-02 test (preferably PCR) within the last 12 months or has been diagnosed presumptive positive and has been treated for COVID-19 within the last 12 months; (3) not fully recovered from COVID-19 in weeks or months despite a negative SARS-COV-02 test and has been diagnosed with post-COVID-19 syndrome; (4) two or more symptoms for over 12 weeks either continually or intermittently with relapses not experienced pre-illness, that interferes with normal daily activities including extreme fatigue, shortness of breath, cough, brain fog, headache, body aches, joint/chest pain, sleep issues, loss of taste/smell; (5) must have previously banked cells at Hope Biosciences, LLC.; (6) Subject and sexual partner (if woman of childbearing potential) must use a highly effective form of birth control throughout the study and for 6 months after the last dose of the investigational product.

#### Exclusion criteria

(1) inability to understand and provide signed informed consent; (2) currently diagnosed with active COVID-19 disease; (3) unwilling to agree to the use of acceptable methods of contraception; (4) pregnancy or breast-feeding women; (5) alcoholic addiction or dependency, substance use or abuse; (6) any active malignancy, including evidence of cutaneous basal, squamous cell carcinoma, or melanoma; (7) one or more significant concurrent medical conditions, including but not limited to poorly controlled diabetes mellitus, chronic kidney disease, cardiac failure, hypertension (> 180/120 mm/Hg), inherited thrombophilia, recent major general surgery, lower extremity paralysis due to spinal cord injury, fracture of the pelvis, hips, femur, cancer of the lung, brain, lymphoma, gynecologic system (ovary or uterus), or gastrointestinal tract (like pancreas or stomach); (8) received any stem cell treatment within 12 months before first dose of investigational product; (9) any laboratory abnormalities including, white blood cell count < 3000/mm^3^, platelet count < 125,000mm^3^, absolute neutrophil count < 1500/mm^3^, alanine aminotransferase (ALT) or aspartate aminotransferase (AST) > upper limit of normal (ULN) × 1.5, or any other laboratory abnormality that poses a safety risk; (10) subject is unlikely to complete the study or adhere to the study procedures; (11) a preexisting lung condition such as chronic obstructive pulmonary disease (COPD); (12) HIV, Hepatitis B or C infection at screening; (13) previously diagnosed psychiatric condition which may affect self-assessments.

### HB-adMSC production

#### Isolation and expansion of autologous HB-adMSCs

For the isolation of HB-adMSCs from each participant, adipose tissue was extracted via liposuction by a licensed physician from the patients’ abdomen. The extract was then delivered for testing by the quality unit at Hope Biosciences LLC., for USP71 sterility and mycoplasma due to possible contamination contracted during the fat extraction procedures, after which the adipose tissue phase was separated by centrifugation. The adipose tissues were then treated with collagenase to enzymatically isolate stromal vascular fraction (SVF). The SVF was then plated in Hope Biosciences’ HB-103 medium to establish a P0 culture. The resulting adherent cells were further cultured with HB-101, Hope Biosciences’ growth medium. The MSCs were cryopreserved at passages 0, 1 and 2 to create a complete bank for individual participant for future autologous use (Additional file 1: Fig. S1). Each lot passed cGMP compliant quality control standard assessments that included cell viability, USP71 sterility, mycoplasma, and endotoxin and cell identity/purity defined by MSC surface markers (Additional file 1: Table S1).

#### HB-adMSC administration

All infusion products prepared for the program were autologous, which were freshly manufactured out of the participant’s own cell bank. Passage 2 cells were thawed, recovered in passage 3, and cultured to passage 4. Each study participant was given a total of five infusions each of 2 × 10^8^ live cells of HB-adMSCs for the treatment duration of 14 weeks that included Infusion 1 (Week 0), Infusion 2 (Week 2), Infusion 3 (Week 6), Infusion 4 (Week 10) and Infusion 5 (Week 14). 2 × 10^8^ ± 20% HB-adMSCs were mixed into a 250 mL bag of 0.9% sterile sodium chloride and then administered intravenously at 83.3 gtts/min for an hour. Clinical monitoring of safety parameters was done for 24 h following each infusion at weeks 0, 2, 6, 10, and 14.

### Study endpoints

The primary objective of the expanded access protocol was to evaluate the efficacy of HB-adMSCs in improving the signs and symptoms associated with post-COVID-19 syndrome. The primary endpoints were assessed using total VAS scores at each visit as well as by assessing subject’s energy and stamina as evidenced on FAS. Secondary efficacy endpoint was to evaluate improvements in the quality-of-life of subjects with post-COVID-19 syndrome, assessed by SF-36 health survey questionnaire. Safety endpoint assessments included clinically significant changes in laboratory values (hematologic, renal, and hepatic), vital signs, weight, and physical examination results.

#### Visual analog scale (VAS)

VAS was used as a psychometric measuring scale to document the characteristics of disease-related symptom severity in individual patients. The objective of the scale was to achieve a rapid (statistically measurable and reproducible) classification of symptom severity and disease control in patient population [26]. Using VAS, we aimed to assess neurological (fatigue, headache, sleep issues, brain fog, loss of taste/smell) and non-neurological (dyspnea, cough, body aches, joint pain) symptoms on the scale from zero to ten centimeters, with zero representing no symptoms and ten describing the worst symptoms possible.

#### Fatigue assessment scale (FAS)

To assess the symptoms of chronic fatigue in an individual, a ten-item scale FAS was used. The FAS considers fatigue as a unidimensional concept rather than a collection of variables that may be measured separately. Additionally, to guarantee that the scale would assess all elements of fatigue, questions that represented both physical and mental symptoms of the disease were included in the scale by the developers [27].

#### Short form questionnaire-36 (SF-36)

The SF-36 is a multi-purpose survey intended to collect patients’ views of their health and well-being. SF-36 consists of 36 questions, organized into eight dimensions: physical functioning, physical and emotional limitations, social functioning, physiological discomfort, general and mental health.

### Statistical analysis

All the results were analyzed using SAS program version 9.4 M7. Data for primary and secondary efficacy were analyzed using pair-wise comparison of baseline and post-treatment assessments of VAS, FAS, and SF-36 scores, using Wilcoxon signed-rank test. The primary goal of the expanded access program was overall comparison before and after HB-adMSC therapy, so no multiplicity adjustments were performed. All continuous variables were presented as median and inter-quartile range (IQR) values. The categorical variables were described using number (N) and percentages based on N. Statistical significance for all primary and secondary outcomes was determined by two-tailed *P*-value < 0.05 with 95% confidence interval (CI). Data for safety analysis included descriptive summary of laboratory evaluations as well as adverse events (AEs) displayed in terms of the number of events (E) and percentage of subjects with at least one event.

To determine the clinical significance for efficacy data, effect sizes were reported. The effect size was calculated for the *P*-values, using Rosenthal’s formula: ES $=\frac{z}{\sqrt{N}}$ where N is the number of subjects [28, 29] and interpreted as small (≥ 0.2), medium (≥ 0.4) and large (≥ 0.8).

## Results

### Demographics and baseline characteristics

**Fig. 1:**
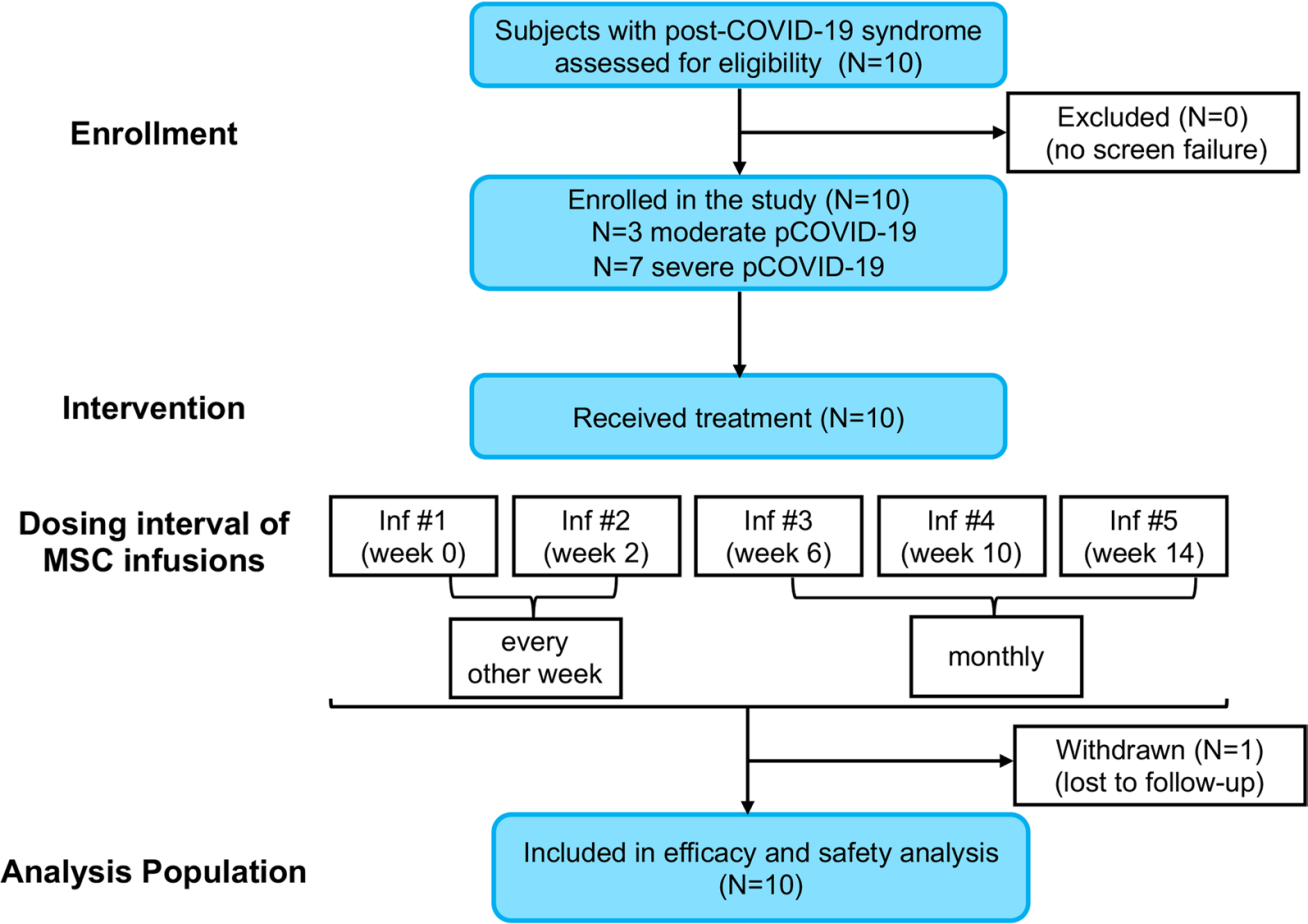
Flow diagram of the study participants. Abbreviations: pCOVID-19, post-Coronavirus disease-2019; Inf, infusion

A total of 10 eligible subjects with post-COVID-19 syndrome were screened and enrolled in the program (no screen failures). Selection of subjects was based on inclusion and exclusion criteria and only those who met all of the inclusion criteria and none of the exclusion criteria, were eligible to participate in this program. Of the 10 subjects exposed to the treatment drug (2 × 10^8^ HB-adMSCs), only one was withdrawn due to lost to follow-up (Fig. 1). The study population consisted of equal percentage of male (50%) and female subjects (50%) with median (range) age of 40.5 (28.0, 61.0) years and was comprised of 100% non-Hispanic ethnicity (Table 1). More detailed information about baseline characteristics including patients’ symptoms and disease severity is provided in Additional file 1: Table S2. Also, a summary of medical history for study subjects is provided in Additional file 1: Table S3.

**Table 1:** Demographic and baseline characteristics of N = 10 subjects

Age (years)	40.5 (28.0, 61.0)
Height (cm)	177.8 (162.6, 190.5)
Weight (kg)	78.9 (50.3, 117.9)
BMI (kg/m^2^)	27.4 (19.0, 43.3)
Sex	
Female	5 (50%)
Male	5 (50%)
Ethnicity	
Hispanic or Latino	0 (0%)
Not Hispanic or Latino	10 (100%)
Race	
American Indian or Alaska Native	0 (0%)
Asian	3 (30%)
Black or African American	1 (10%)
Native Hawaiian or another Pacific Islander	0 (0%)
White	6 (60%)

*BMI* body mass index; statistics represented: median (range); N (%)

### Efficacy evaluation

The effectiveness of HB-adMSCs was assessed through improvements in VAS scores as well as FAS scores, measured before and after-treatment, at each visit. Also, to evaluate improvements in the quality-of-life of the subjects, Short Form-36 health survey questionnaire was assessed.

#### Visual analog scale (VAS) and fatigue assessment scores (FAS)

**Fig. 2:**
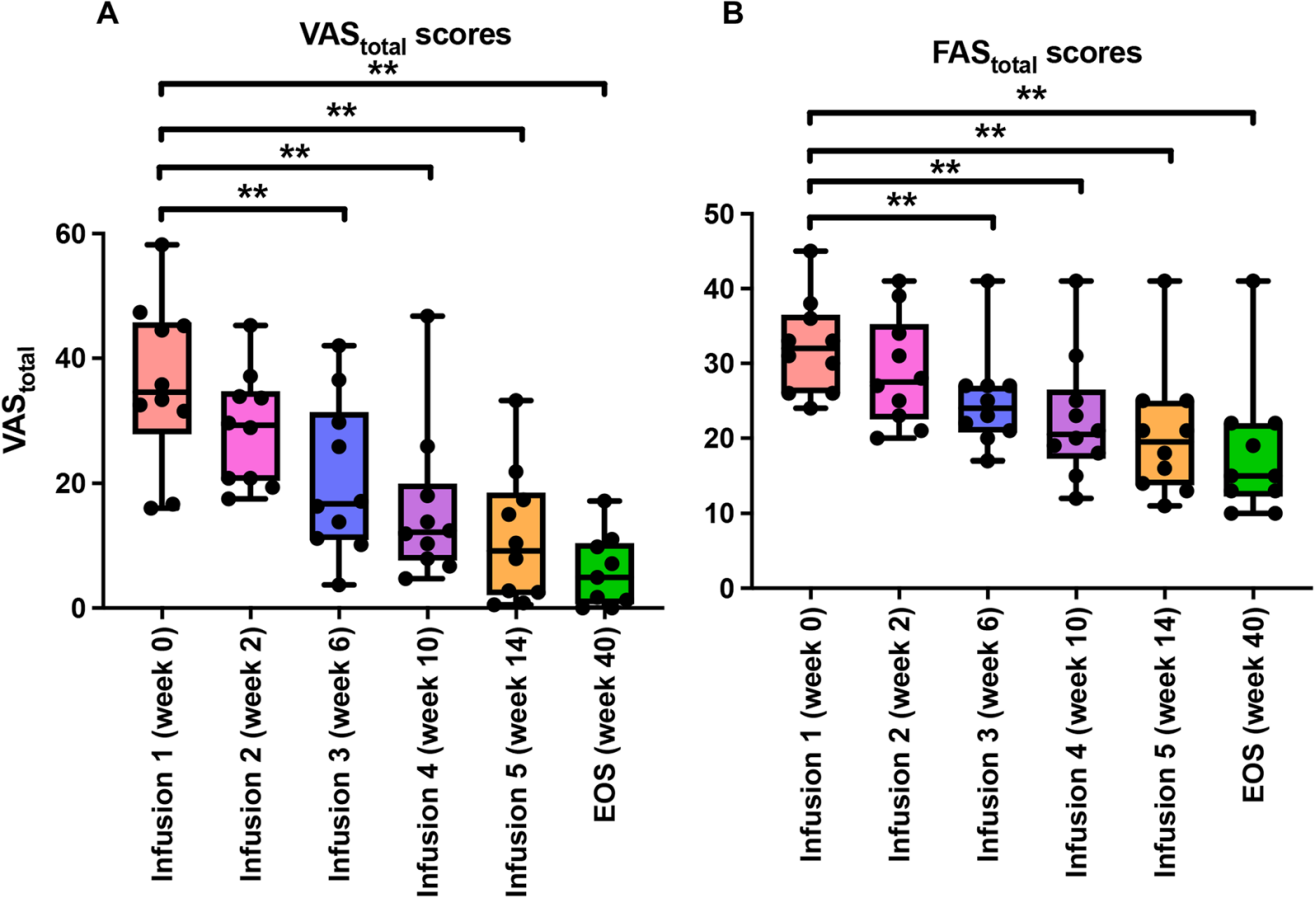
VAS_total_ and FAS_total_ scores. Both VAS_total_ and FAS_total_ scores showed significant declines apparent as early as infusion #3 through EOS post-treatment, compared to baseline. Significance defined at *P*-value < 0.05, **P-value < 0.01 (Wilcoxon signed-rank test). Abbreviation: EOS, end of study

Both VAS_total_ and FAS_total_ scores demonstrated significant improvements at the end of the study period compared to baseline (week 0, Infusion#1), with the median values declining from 37.6 (IQR 33.1–44.3) to 4.90 (IQR:1.3–9.8) for VAS_total_ scores (*P*-value 0.0039) and from 32.5 (IQR:27.0–35.0) to 15.0 (IQR:13.0–19.0) for FAS_total_ score (*P*-value 0.0039), respectively, with large effect sizes (ES = 0.91 for both for VAS_total_ and FAS_total_) (Fig. 2 A&B, Table 2).

**Table 2:** Efficacy measures for total VAS and FAS scores at baseline and EOS post-treatment

	Baseline Median (IQR)	EOS Median (IQR)	*P*-value	Effect size (ES)
VAS_total_	37.6 (33.1–44.3)	4.90 (1.26–9.81)	0.0039**	0.91
FAS_total_	33.0 (27.0–35.0)	15.0 (13.0–19.0)	0.0039**	0.91

*VAS* visual analog scale, *FAS* Fatigue Assessment Score, *EOS* end of study, *IQR* inter-quartile range (25–75%)

^**^P < 0.01 (Wilcoxon signed-rank test). ES = Effect Size (Rosenthal’s formula with N = 10 subjects)

#### SF-36 questionnaire

**Fig. 3:**
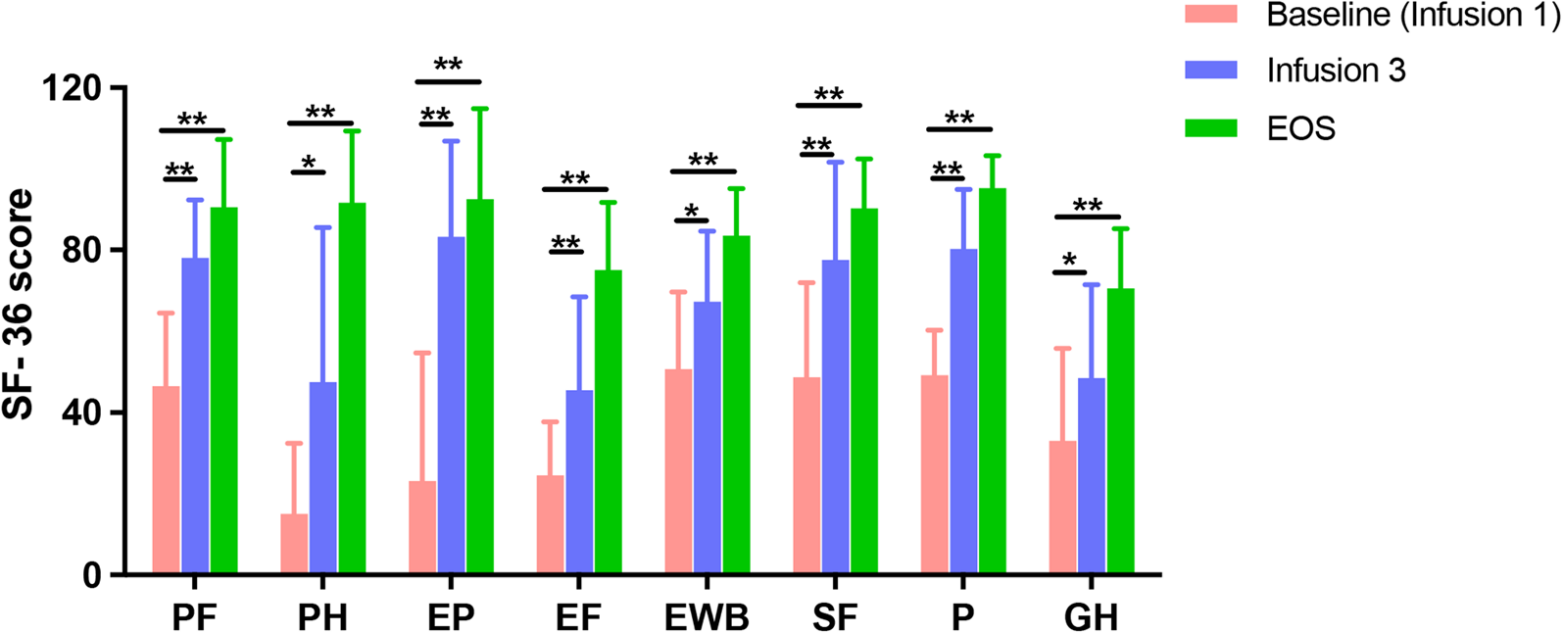
SF-36 scores. Individual SF-36 scaled scores at baseline (infusion #1), at week 6 (Infusion #3) and at EOS. Comparison between baseline and EOS scores demonstrated significant increases in all individual scores; *P-value < 0.05, **P-value < 0.01 (Wilcoxon signed-rank test)

The SF-36 health questionnaire consists of eight scaled scores: Energy/Fatigue scores (EF), Social Functioning (SF), role limitations due to Physical Health (PH), General Health (GH), Physical Functioning (PF), Pain (P), Emotional Well-Being (EWB), and Emotional Problems (EP) which are the weighted sums of the questions per section (on a scale of 0–100; higher score indicates lower disability). Paired comparison between baseline and EOS SF-36 scores showed improved quality-of-life with significant changes in individual SF-36 evaluations (*P*-value = 0.0039 and ES = 0.91 for PF, EF, EWB, P and PH; *P*-value = 0.0078 and ES = 0.84 for EP, GH, and SF) (Fig. 3, Table 3).

**Table 3:** Individual SF-36 scores at baseline and EOS post-treatment

SF-36 scales	Baseline Median (IQR) N = 10	EOS Median (IQR) N = 9	*P*-value	Effect size (ES)
PF	42.5 (35.0–55.0)	100.0 (90.0–100.0)	0.0039**	0.91
PH	12.5 (0.00–25.0)	100.0 (100.0–100.0)	0.0039**	0.91
EP	16.7 (0.00–33.3)	100.0 (100.0–100.0)	0.0078**	0.84
E/F	25.0 (25.0–35.0)	80.0 (75.0–80.0)	0.0039**	0.91
EWB	58.0 (36.0–72.0)	88.0 (84.0–92.0)	0.0039**	0.91
SF	50.0 (25.0–62.5)	100.0 (75.0–100.0)	0.0078**	0.84
P	45.0 (45.0–67.5)	100.0 (90.0–100.0)	0.0039**	0.91
GH	32.5 (10.0–50.0)	70.0 (60.0–80.0)	0.0078**	0.84

*PF* role limitations due to physical functioning, *PH* role limitations due to physical health, *EP* role limitations due to emotional problems, *E/F* energy/fatigue, *EWB* emotional well-being, *SF* social functioning, *P* pain, *GH* general health, *N* number of subjects, *EOS* end of study, *IQR* inter-quartile range

^**^P < 0.01 (Wilcoxon signed-rank test). ES = Effect Size (Rosenthal’s formula with N = 10 subjects)

### Safety evaluation

#### Safety and tolerability

Standard laboratory evaluations included hematologic measures, biochemistry laboratory panel, and coagulation tests that were evaluated at the baseline and at the end of the study (EOS). No clinically relevant changes were observed at the EOS in any of metabolic or biochemistry parameters compared to baseline (*P* > 0.05). Coagulation panel measures also remained largely unchanged at the EOS compared to baseline values. Descriptive statistics for all laboratory results for safety measures are summarized in Additional file 1: Table S4.

#### Adverse events (AEs)

A total of 40 adverse events (AEs) were recorded during the entire course of study, out of which 39 (97.5%) were mild in severity, only one (0.03%) was moderate, and none (0%) was severe. Almost half of the reported AEs were considered unlikely or unrelated to the investigational drug. All five infusions were well tolerated by all subjects with no occurrence of any serious adverse events (SAEs). No deaths were reported during the entire treatment period. Headaches and influenza-like illness accounted for 30/40 (75%) of the AEs while the remaining 10 AEs included disorders like diarrhea, nausea, myalgia, sinus, etc. (Additional file 1: Table S5).

## Discussion

Due to the uncontrolled inflammatory response associated with COVID-19, several affected COVID-19 patients passively develop long-term sequalae, immune disruptions, and even severe complications, like ARDS [30, 31]. Despite multiple strategies [8, 9, 11] that have been developed to combat COVID-19 and its associated complications, there is still a need for a safe and effective therapeutic to help resolve the post-COVID-19 sequelae. Based on their immunomodulatory, anti-inflammatory, and regenerative properties, MSCs have emerged as a potentially safe and effective therapeutic option to treat COVID-19 and its long-term consequences. The current study employed multiple intravenous infusions of autologous, adipose-derived MSCs with a goal to improve signs and symptoms associated with post-COVID-19 syndrome. To the best of our knowledge, this is the first study that used adipose-derived MSCs to evaluate safety and efficacy of MSCs over a period of 40 weeks to treat COVID-19 long-haulers.

Post-SARS-CoV-2 infection, the long-haulers present significantly impaired quality-of-life (QoL) and suffer from persistent symptoms dominated by fatigue, shortness of breath, cough, body aches, joint pain, brain fog, among others. The low QoL merits safe and effective treatment. The results of the efficacy assessments (VAS, FAS and SF-36 scores) in the current study indicated improvements in clinical symptoms (with large effect sizes) at the EOS compared to baseline. Almost all patients regained functionality with significant improvements in the QoL. Assessments of total VAS scores as well as total FAS scores used to evaluate subject’s energy and stamina, demonstrated clinically relevant improvements at the EOS vs baseline. Also, the study employed SF-36 health questionnaire to evaluate QoL of COVID-19 survivors with post-COVID-19 syndrome, post-treatment with HB-adMSC therapy. Efficacy data analysis revealed statistically significant improvements in almost each individual symptom. We found significant increases in all the individual SF-36 scales (role limitations due to physical functioning; role limitations due to physical health; role limitations due to emotional problems; energy/fatigue; emotional well-being; social functioning; pain and general health) at the EOS compared to the baseline, implicating improved QoL.

For a more comprehensive efficacy evaluation, studies have evaluated oxygen saturation levels and respiration rates before and after treatment with MSCs. Xu et al. demonstrated significant increases in oxygen saturation after treatment with menstrual blood derived-MSCs [32]. Leng et al. also reported improved oxygen saturation levels resulting in regain of pulmonary function post-MSCs transplantation [33]. But, in the current study, both oxygen saturation levels and respiration rates remained similar to the baseline without any significant changes. It should be noted, however, that these parameters were already within normal range at the baseline.

Safety of MSC transplantation in COVID-19 patients has been demonstrated by several studies [24, 32, 34]. Specifically, safety of adipose-tissue derived MSC therapy for COVID-19 has also been previously reported [35, 36]. In resonance with these studies, we also observed that treatment with multiple intravenous infusions of HB-adMSCs is safe, without any detrimental effects in laboratory or non-laboratory measures.

The current study has several limitations. First, this was a small-sample population expanded access study with no placebo comparator to evaluate. Also, due to small population size, there was lack of ethnic diversity which warrants a larger sample population with a randomized study design that will also help to minimize any selection bias. Second, the inclusion criteria did not define any threshold for oxygen saturation and respiration levels—since all subjects had normal baseline respiration rates and oxygen saturation levels, we were unable to see any possible improvements in pulmonary function that might also be relevant to efficacy outcomes, post-MSC therapy. Third, this was a medium-term, 40-week long study (26-week follow-up post-MSC-infusions). In order to see long-term improvements, longer follow-ups are needed. Fourth, no pre-COVID assessments were obtained on any of the efficacy evaluations which could have helped determine what extent of health and functionality got recovered post-MSC treatment.

## Conclusions

In conclusion, treatment of patients with post-COVID-19 syndrome using multiple infusions of fresh, autologous, HB-adMSCs not only presented beneficial effects in improving and reversing associated signs and symptoms, but also implicated therapeutic sustainability over a period of 40 weeks. Findings of the current study presented a strong suggestion of amelioration of clinical symptoms associated with post-COVID-19 syndrome, following HB-adMSC therapy. Safety evaluation also demonstrated good tolerability with only mild AEs and no incidence of SAEs. The results of the current expanded access program should be confirmed with a larger, placebo-controlled randomized trial with a longer follow-up.

### Supplementary Information

**Additional file 1**: MSC quality control metrics for all five infusions for N=10 subjects are given in Table S1. Adipose tissue extraction and expansion is provided in Figure S1. Baseline characteristics for N=10 subjects are provided in Table S2 and summary of medical history for all 10 subjects is provided in Table S3. Summary statistics for all safety laboratory parameters are provided in Table S4. Summary of adverse events by reported term for all subjects is provided in Table S5 in Additional file 1.

## Data Availability

The data that support the findings of this expanded access program are available on request by emailing the corresponding author.

## Abbreviations

adMSCs: Adipose-derived Mesenchymal Stem Cells
AE: Adverse Event
ARDS: Acute Respiratory Distress Syndrome
CI: Confidence Interval
COVID-19: CoronaVirus Disease-2019
EOS: End of Study
ES: Effect Size
FAS: Fatigue Assessment Score
MSCs: Mesenchymal Stem Cells
SARS-CoV-2: Severe Acute Respiratory Syndrome Coronavirus 2
SAE: Serious Adverse Event
SF-36: Short Form-36
VAS: Visual Analog Scale
WIRB: Western Institutional Review Board
