# Supplementary material for: Adipose-derived, autologous mesenchymal stem cell therapy for patients with post-COVID-19 syndrome: an intermediate-size expanded access program
Source: Stem Cell Res Ther. 2023 Oct 5;14:287. doi: 10.1186/s13287-023-03522-1 (PMC10557203; doi:10.1186/s13287-023-03522-1)
Supplement: Supplementary file 1 — Additional file 1: MSC quality control metrics for all five infusions for N=10 subjects are given in Table S1. Adipose tissue extraction and expansion is provided in Figure S1. Baseline characteristics for N=10 subjects are provided in Table S2 and summary of medical history for all 10 subjects is provided in Table S3. Summary statistics for all safety laboratory parameters are provided in Table S4. Summary of adverse events by reported term for all subjects is provided in Table S5 in Additional file 1. [file 13287_2023_3522_MOESM1_ESM.docx]

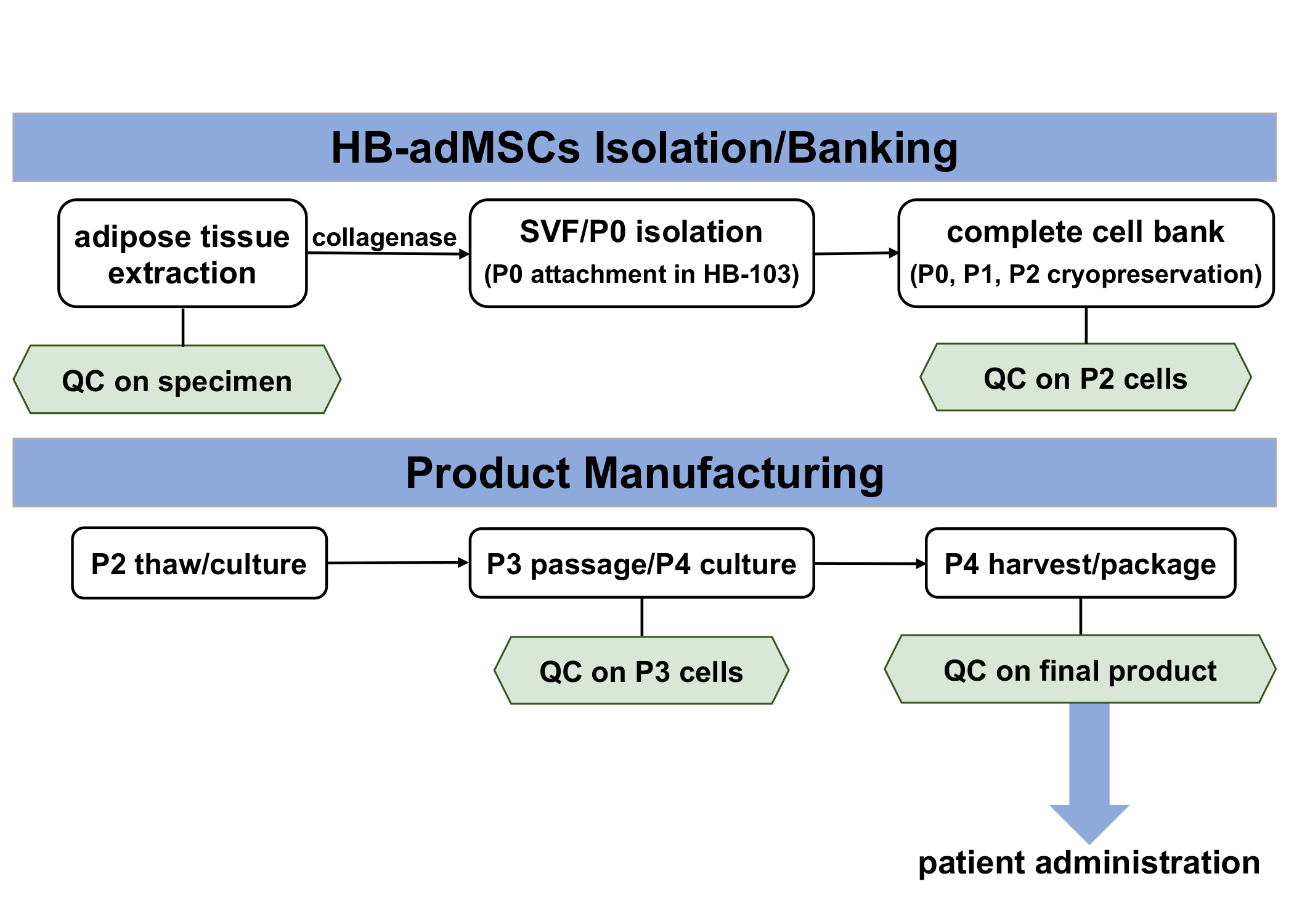


**Figure S1. Adipose tissue extraction and expansion.** Adipose tissue was extracted via liposuction and complete cell bank was created for each subject by cryopreserving P0, P1, and P2 of HB-adMSCs. Before a scheduled administration, vial(s) of P2 cells were thawed out to culture P3 cells, which were further expanded to culture P4. The drug (harvested P4 cells) was packaged into a syringe for patient administration. adMSCs were cultured using HB-101, a proprietary growth medium of Hope Biosciences, across all passages.

**Supplementary Table Legends**

**Table S1. MSC quality control metrics for all five infusions (N=10 subjects)**

*Technician error in cell counting procedure caused low cell-count. ** Technician error in cell harvesting procedure caused low cell-count. MSCs are expected to be positive for CD73 and CD29 and negative for CD45 and CD31 surface markers.

**Table S2. Baseline characteristics for N=10 subjects**

*****Subject unsure of the exact dates. Abbreviations, SOB, shortness of breath; pCOVID-19, post-COVID-19; mod, moderate; sev, severe

**Table S3. Summary of medical history for N=10 subjects**

**Table S4. Summary statistics of all safety laboratory parameters**

Summary evaluation of lab values at the baseline and at end of study. p-values calculated using Wilcoxon test by comparing EOS pair-wise with baseline.

**Table S5. Adverse events by reported term for all 10 subjects**

*Subject HBPCOV01-01-004 had a pre-existing condition of benign mediastinal mass that was not previously biopsied by recommendation of his oncologist and the subject was cleared to be included in the study. It was biopsied after treatment and found to be follicular lymphoma.

**Table S1. MSC quality control metrics for all five infusions (N=10 subjects)**

|  | **Infusion #** | **Total cell count (million)** | **Cell viability**  **(%)** | **CD73**  **(%)** | **CD29**  **(%)** | **CD31**  **(%)** | **CD45**  **(%)** |
| --- | --- | --- | --- | --- | --- | --- | --- |
| **Subject #1** | 1  2  3  4  5 | 240  240  240  240  240 | 97.41  96.00  97.73  94.34  97.35 | 83.14  82.67  98.83  97.22  96.34 | 100.0  96.05  100.0  99.0  99.38 | 0.00  0.24  0.03  0.00  0.07 | 0.00  0.05  0.00  0.07  0.23 |
| **Subject #2** | 1  2  3  4  5 | 240  240  240  240  240 | 94.51  94.06  9505  97.09  97.90 | 90.48  76.01  84.95  84.79  85.22 | 99.96  99.92  100.0  99.89  99.01 | 3.11  0.00  0.00  0.00  0.04 | 0.15  0.00  0.12  0.30  0.07 |
| **Subject #3** | 1  2  3  4  5 | 240  166  240  214  240 | 96.30  92.86  94.12  95.71  97.47 | 87.98  92.34  95.87  93.29  94.89 | 94.86  99.53  99.89  99.26  99.83 | 0.42  0.12  0.00  0.00  0.00 | 0.13  0.04  0.11  0.20  0.07 |
| **Subject #4** | 1  2  3  4  5 | 240  240  176  240  240 | 97.85  93.59  96.49  98.68  97.12 | 93.34  93.81  94.45  99.55  95.23 | 99.78  99.86  99.65  99.90  99.70 | 0.00  0.00  0.04  0.00  0.04 | 0.29  0.21  0.25  0.20  0.27 |
| **Subject #5** | 1  2  3  4  5 | 240  240  240  240  240 | 99.02  98.26  98.08  97.98  96.77 | 89.26  81.43  92.24  91.61  91.86 | 99.62  95.60  99.53  99.90  99.96 | 0.00  0.04  0.00  0.00  0.00 | 0.15  0.00  0.06  0.13  0.04 |
| **Subject #6** | 1  2  3  4  5 | 240  192  240  240  240 | 92.55  95.24  98.88  97.79  97.46 | 92.53  94.95  76.94  96.46  86.94 | 99.56  99.74  99.73  99.94  99.88 | 0.00  0.03  0.00  0.00  0.03 | 0.20  0.03  0.03  0.10  0.00 |
| **Subject #7** | 1  2  3  4  5 | 218  120*  195  240  211 | 97.14  93.75  98.39  94.19  95.65 | 91.40  95.53  78.74  94.36  88.79 | 99.87  95.56  98.39  99.76  99.96 | 0.03  0.11  0.00  0.00  0.00 | 0.14  0.22  0.06  0.52  0.12 |
| **Subject #8** | 1  2  3  4  5 | 238  224  211  208  240 | 93.13  98.59  94.29  97.01  96.59 | 90.86  94.68  99.16  89.80  91.43 | 99.73  99.05  99.82  99.69  99.93 | 0.00  0.08  0.00  0.00  0.02 | 0.03  0.27  0.04  0.09  0.02 |
| **Subject #9** | 1  2  3  4  5 | 240  240  237  240  240 | 96.58  94.19  96.10  93.75  99.15 | 94.38  91.89  98.43  92.81  93.93 | 99.68  99.54  99.84  99.66  100.0 | 0.00  0.04  0.05  0.00  0.00 | 0.16  0.12  0.05  0.22  0.02 |
| **Subject #10** | 1  2  3  4  5 | 240  150**  192  234  240 | 95.00  97.92  96.77  97.33  93.22 | 97.94  99.38  95.65  96.03  92.63 | 98.95  99.94  99.43  100.0  100.0 | 0.00  0.06  0.00  0.03  0.00 | 0.13  0.11  0.32  0.20  0.00 |

**Table S2. Baseline characteristics for N=10 subjects**

| **#** | **Age**  **(years)** | **Gender**  **(M/F)** | **Smoking**  **status (Yes/No)** | **Suspected COVID-19**  **(Start date/**  **End date)** | **Severity of**  **COVID-19** | **pCOVID-19**  **start dates** | **pCOVID-19**  **symptoms** | **Severity of pCOVID-19 symptoms** | **Date of First Infusion (HB-adMSCs)** |
| --- | --- | --- | --- | --- | --- | --- | --- | --- | --- |
| **1** | 61 | F | No | 01-X-2020/  01-X-2020* | mod | 01-X-2020* | fatigue, recurrent bronchitis, chest pain, joint pain | sev | 03-23-2021 |
| **2** | 45 | M | No | 07-X-2020/  07-X-2020* | mod | 07-X-2020* | fatigue, brain fog, anosmia, ageusia, headache | mod | 04-01-2021 |
| **3** | 38 | F | No | 07-09-2020/  08-21-2020 | mod | 08-X-2020* | fatigue, body aches, tingling | mod | 04-06-2021 |
| **4** | 61 | M | Yes | 12/10/2020/  10-22-2020 | mod | 03-12-2021 | SOB, fatigue, mild anosmia, ankle and knee pain | sev | 05-18-2021 |
| **5** | 28 | M | No | 06-20-2020/  06-29-2020 | sev | 06-29-2020 | fatigue, brain fog, decreased memory | sev | 06-02-2021 |
| **6** | 35 | F | Yes | 04-09-2020/  06-07-2020 | mod | 04-18-2020 | chronic fatigue, low grade fever, dizziness, nausea, insomnia | sev | 06-02-2021 |
| **7** | 32 | M | No | 08-01-2020/  08-15-2020 | sev | 08-15-2020 | brain fog, blurry vision, dysautonomia, neuropathy, tachycardia, SOB, decreased energy, fatigue, light sensitivity | sev | 06-29-2021 |
| **8** | 39 | M | No | 06-20-2020/  06-29-2020 | mod | 06-29-2020 | SOB with wheezes, fatigue, body aches, arthralgia | sev | 06-02-2021 |
| **9** | 42 | F | No | 08-03-2020/  08-17-2020 | sev | 08-17-2020 | fatigue, dizziness, brain fog, SOB, depression, anxiety | sev | 06-15-2021 |
| **10** | 51 | F | No | 12-28-2020/01-10-2021 | mod | 01-11-2021 | anosmia, ageusia, fatigue, dizziness, body aches | mod | 06-15-2021 |

**Table S3. Summary of medical history for N=10 subjects**

|  | **N (%)** |
| --- | --- |
| **Subjects with Medical History** | **10 (100.0)** |
| Fatigue | 10 (100.0) |
| Clouded consciousness | 7 (70.0) |
| Dyspnea | 7 (70.0) |
| Generalized aches and pains | 6 (60.0) |
| Joint pain | 6 (60.0) |
| Seasonal allergy | 6 (60.0) |
| Chronic headache disorder | 5 (50.0) |
| Loss of hair | 5 (50.0) |
| Asthma | 4 (40.0) |
| Depressive disorder | 4 (40.0) |
| Dizziness | 3 (30.0) |
| Irregular periods | 3 (30.0) |
| Loss of sense of smell | 3 (30.0) |
| Obesity | 3 (30.0) |
| Anxiety | 2 (20.0) |
| Cesarean section | 2 (20.0) |
| Chest pain | 2 (20.0) |
| Chronic constipation | 2 (20.0) |
| Disorder of autonomic nervous system | 2 (20.0) |
| Gastric sleeve | 2 (20.0) |
| Gastroesophageal reflux disease | 2 (20.0) |
| Hernia of abdominal cavity | 2 (20.0) |
| Hypercholesterolemia | 2 (20.0) |
| Influenza | 2 (20.0) |
| Loss of taste | 2 (20.0) |
| Abnormal cervical Papanicolaou smear | 1 (10.0) |
| Abnormal vaginal Papanicolaou smear | 1 (10.0) |
| Aphasia | 1 (10.0) |
| Arthroscopy of knee | 1 (10.0) |
| Basal cell carcinoma of nose | 1 (10.0) |
| Basal cell carcinoma of scalp | 1 (10.0) |
| Basal cell carcinoma of upper back | 1 (10.0) |
| Basal cell carcinoma of upper extremity | 1 (10.0) |
| Benign prostatic hyperplasia | 1 (10.0) |
| Bipolar | 1 (10.0) |
| Blurring of visual image | 1 (10.0) |
| Cerebrovascular accident | 1 (10.0) |
| Chronic bronchitis | 1 (10.0) |
| Dilation and curettage | 1 (10.0) |
| Disorder of lung | 1 (10.0) |
| Dyslipidemia | 1 (10.0) |
| Eczema | 1 (10.0) |
| Endometrial ablation | 1 (10.0) |
| Endometriosis | 1 (10.0) |
| Excision of bunion | 1 (10.0) |
| Hazy vision | 1 (10.0) |
| Hemorrhoids | 1 (10.0) |
| Herpes zoster | 1 (10.0) |
| Hypertensive disorder | 1 (10.0) |
| Hyperthyroidism | 1 (10.0) |
| Hypothyroidism | 1 (10.0) |
| Hysterectomy | 1 (10.0) |
| Infertility due to systemic disease | 1 (10.0) |
| Insomnia | 1 (10.0) |
| Iron deficiency anemia | 1 (10.0) |
| Itching of skin | 1 (10.0) |
| Low grade pyrexia | 1 (10.0) |
| Lumbar radiculopathy | 1 (10.0) |
| Malaise | 1 (10.0) |
| Melanoma in situ of back | 1 (10.0) |
| Mixed hyperlipidemia | 1 (10.0) |
| Mycosis | 1 (10.0) |
| Myocarditis | 1 (10.0) |
| Nausea | 1 (10.0) |
| Neuropathy | 1 (10.0) |
| Obsessive-compulsive disorder | 1 (10.0) |
| Oophorectomy | 1 (10.0) |
| Photophobia | 1 (10.0) |
| Sciatica | 1 (10.0) |
| Sinus tachycardia | 1 (10.0) |
| Small fiber neuropathy | 1 (10.0) |
| Steatosis of liver | 1 (10.0) |
| Sweating | 1 (10.0) |
| Tachycardia | 1 (10.0) |
| Thyroid gland ablation - irradiation | 1 (10.0) |
| Tinnitus | 1 (10.0) |
| Tonsillectomy | 1 (10.0) |
| Total knee replacement | 1 (10.0) |
| Type 2 diabetes mellitus | 1 (10.0) |
| Uterine leiomyoma | 1 (10.0) |
| Vasovagal syncope | 1 (10.0) |

**Table S4. Summary statistics of all safety laboratory parameters**

| **Variable** | | **Baseline** | | | **EOS** | **p-value** |
| --- | --- | --- | --- | --- | --- | --- |
|  |  | **Median (IQR 25^th^-75^th^)** | | | **Median (IQR 25^th^-75^th^)** |  |
|  |  | **N=10** | | | **N=9** |  |
| **Biochemistry** | |  | | |  |  |
| Alkaline Phosphatase (IU/L) | | 65.0 (56.0-82.0) | | | 73.0 (62.0-83.0) | 0.418 |
| Alanine Aminotransferase (IU/L) | | 21.5 (17.0-27.0) | | | 20.0 (18.0-22.0) | 0.281 |
| Aspartate Aminotransferase (IU/L) | | 23.5 (20.0-26.0) | | | 22.0 (17.0-33.0) | 0.516 |
| Calcium (mg/dL) | | 9.8 (9.5-9.9) | | | 9.6 (9.4-9.7) | 0.375 |
| Bilirubin (mg/dL) | | 0.5 (0.2-1.2) | | | 0.6 (0.5-1.2) | 0.375 |
| Chloride (mmol/L) | | 103.0 (102.0-105.0) | | | 103.0 (102.0-103.0) | 0.625 |
| Carbon Dioxide (mmol/L) | | 25.5 (25.0-27.0) | | | 25.0 (23.0-25.0) | 0.125 |
| Creatinine (mg/dL) | | 0.82 (0.76-1.0) | | | 0.84 (0.74-1.01) | 0.882 |
| C-Reactive Protein (mg/L) | | 1.40 (1.1-2.3) | | | 3.5 (3.5-3.5) | 1.000 |
| Glucose (mg/dL) | | 92.5 (88.0-100.0) | | | 109.0 (90.0-128.0) | 0.262 |
| Potassium (mmol/L) | | 4.2 (4.1-4.2) | | | 4.2 (4.1-4.4) | 0.305 |
| Protein (g/dL) | | 7.0 (6.8-7.5) | | | 7.2 (6.9-7.4) | 0.793 |
| Sodium (mmol/L) | | 141.5 (140.0-143.0) | | | 142 (139.0-142.0) | 0.113 |
| Urea Nitrogen (mg/dL) | | 14.0 (14.0-16.0) | | | 14.0 (13.0-17.0) | 0.063 |
|  |  | |  |  |  |  |
| **Hematologic Measures** | |  | | |  |  |
| Basophils (%) | | 0.0 (0.0-0.8) | | | 0.9 (0.8-0.9) | 0.179 |
| Eosinophils (%) | | 1.3 (0.8-3.4) | | | 2.02 (0.0-2.6) | 0.438 |
| Erythrocyte Sedimentation Rate (mm/h) | | 2.0 (1.0-6.0) | | | 2.0 (2.0-5.0) | 0.281 |
| Hematocrit (%) | | 41.0 (40.3-42.0) | | | 41.1 (34.8-42.5) | 0.547 |
| Hemoglobin (g/dL) | | 14.2 (13.1-14.5) | | | 14.2 (13.6-14.6) | 1.000 |
| Lymphocytes (%) | | 34.5 (25.6-40.6) | | | 29.8 (24.3-35.1) | 0.820 |
| Ery. Mean Corpuscular Volume (fL) | | 88.9 (85.0-90.8) | | | 88.4 (84.9-91.4) | 0.398 |
| Monocytes (%) | | 7.7 (5.5-8.6) | | | 6.10 (4.9-7.0) | 0.734 |
| Neutrophils (%) | | 53.9 (49.0-69.3) | | | 62.3 (56.0-66.9) | 0.652 |
| Platelets (10^9/L) | | 282.0 (204.0-311.0) | | | 278.0 (216.0-336.0) | 0.098 |
| Erythrocytes (10^12/L) | | 4.64 (4.45-4.86) | | | 4.59 (4.50-4.65) | 0.816 |
| Leukocytes (10^9/L) | | 6.15 (5.6-8.6) | | | 6.0 (4.9-7.1) | 0.375 |
| Prothrombin Time (s) | | 13.7 (13.3-14.1) | | | 13.2 (12.8-13.5) | 0.109 |
| Partial Thromboplastin Time (s) | | 31.7 (30.2-33.1) | | | 30.3 (28.0-30.7) | 0.125 |

**Table S5. Adverse events by reported term for all 10 subjects**

| **Reported term** | **Adverse Event** | **E** |
| --- | --- | --- |
| **Nervous system disorders** | headache  dizziness | **17**  11  6 |
| **General disorders and administration site conditions** | Influenza-like illness  Pyrexia  Chills | **13**  10  2  1 |
| **Infections and infestations** | COVID-19  Gastroenteritis | **2**  1  1 |
| **Gastrointestinal disorders** | Diarrhea  Nausea | **2**  1  1 |
| **Investigations** | Aspergillus test positive | **1**  1 |
| **Metabolism and nutrition disorders** | Hyperglycemia | **1**  1 |
| **Musculoskeletal disorders** | Myalgia | **1**  1 |
| **Neoplasms benign, malignant, and unspecified** | Follicular lymphoma* | **1**  1 |
| **Respiratory and thoracic disorders** | Sinus congestion | **1**  1 |
| **Skin and subcutaneous tissue disorders** | Erythema | **1**  1 |
